# Selections

**Published:** 1892-11

**Authors:** 


### *SELECTIONS.*

---

### Asepsis and Antisepsis in the Country.

Cleanliness, not in the ordinary sense of the term, but *surgical cleanliness*, is the foundation of asepticism. If we could be sure that everything pertaining to an operation was free from germs, and the field of operation perfectly aseptic, we should have no need of the use of chemical agents as antiseptics. But, as many accidental wounds are septic on account of foreign matter that has got into them at the time of the injury or afterwards by filthy dressings applied before the surgeon arrives, and as operations are often done in regions of the body that are naturally septic, as the mouth, rectum and vagina, we must resort to agents that tend to prevent putrefaction. Surgical instruments sometimes carry septic matter into a wound. They can be sterilized by either dry or moist heat, the latter being the best. They should be very thoroughly scrubbed with soap and water after each operation, and just previous to the operation should be rolled up in a cloth and submitted to the action of steam in a sterilizer for an hour or boiled in water for five or ten minutes. They should then be put into carbolic acid solution (1-20), and kept immersed during the operation. The hands of operator and assistant should be very thoroughly scrubbed with soap and water and then in a saturated solution of permanganate of potash or peroxide of hydrogen. The discoloration of the hands can be removed by a saturated solution of oxalic acid.

A vessel of carbolic acid solution (1-30) should be used during the operation to dip the hands and instruments into. If the field of operation can be planned before hand, it should be shaved and scrubbed thoroughly with soap and water and then with corrosive sublimate (1-500) or peroxide of hydrogen ; and then several layers of aseptic gauze which has been wet in solution of corrosive (1-2000) applied and kept in place by a roller until the time for the operation. Braided silk, for sutures and ligatures, can be boiled and then kept in alcohol. Catgut is not so reliable. It is not easy to keep sponges clean. I make them of wool covered with gauze, and after use throw them away. They should be boiled, dried, and put in glass jars, and just before operation should be again boiled or steamed.

There are many forms of dressing, but I find that what is known as the "baked dressing" is the most convenient to carry around in the country. It is corrosive gauze that contains ten per cent. of glycerine to keep it moist and render it more absorbent. A roll of gauze is rolled up in a sheet of cotton wadding which covers it and keeps it in place. It is put into a tin can and baked in an oven heated to 250° F. The can should not be opened until the dressing is used. In laparotomies long strips of aseptic gauze are excellent to tuck into the abdominal cavity to take up the fluids. In dressings the gauze should be ten or twelve layers thick and should extend a good distance from the wound. The dressing is not cheap. It will save much expense to buy the gauze by the web and prepare it yourself, and you will have a better article.

Needles can be carried in glass ignition tubes with cotton and a cork at the end. These can be held for a moment over an alcohol lamp to render them aseptic.—DR. PROUTY, *College and Clinical Record.*

---

### THE TREATMENT OF LEUCORRHŒA.

Gallois (*Jour. de Med. de Paris*, in *Therapeutic Gazette*) recommends the following solutions:

| | | |
|---|---|---|
| ℞. | Sulphate of copper, . . | gr. xv. |
| | Water, . . . . . . | ℥ viij. |

M. Sig.—Make a solution and use as an injection in chronic leucorrhœa.

Prescribe at the same time ferruginous preparations, internally, and baths.

Another formula which is useful is:

℞. Salicylic acid, . . . . gr. xc.
Glycerine, . . . . . ℥ iv.
Water, . . . . . . Oij.

M. Sig.—Dissolve the salicylic acid in the glycerine by the aid of heat and add water.

This amount is used in six injections, one being given each day in vaginitis due to irritation or inflammation about the uterus or vulva.

Still another formula is one containing:

℞. Chlorate of potassium, . . ʒ iv.

Wine of opium, . . . . ʒ iiss.

Tar-water, . . . . . Oj.

M. Sig.—Make a solution, and in half a pint of hot water place two or three large tablespoonfuls of this solution, and, after mixing thoroughly, employ as an injection in fetid leucorrhœa.

As an astringent injection the following may be employed:

℞. Powdered catechu.
Powdered myrrh, aa gr. lxxv.
Lime-water, ℥ viij.

M. Sig.—Filter after prolonged maceration.

Give this injection several times a day, as long as the necessity continues, or the following may be employed:

℞. Chlorate of potassium, ʒj to ʒiij.

Wine of opium, ʒ iiss.

Tar-water, ℥ viij.

M. Sig.—Make a solution, and add two or three dessert-spoonfuls to a quart of water, employing as an injection night and morning, particularly in cases of leucorrhœa associated with endometritis, polyps or fibroids. The duration of the injection should be about five to six minutes.

In cases of leucorrhœa or vulvitis, occurring in young girls in which there is no true vaginitis or metritis, the inflammation about the vulva is to be combated. This treatment is both local and general. The parts are to be washed carefully with astringent decoctions or with a weak solution of Goulard's extract. After this has been done, the washing may be carried out by weak solutions of bichloride of mercury and by baths and lotions.

In other cases, carbolic acid in the strength of five parts to one thousand of water, may be employed; and finally, in obstinate cases, a solution of nitrate of silver, in the strength of three grains to the ounce, may be resorted to. In the intervals between the bathings, the parts are to be separated by lint impregnated with a weak solution of carbolic acid, or covered with red precipitate ointment. The internal medication consists in the use of cod-liver oil with quinine, or, in scrofulous children, the use of arsenical preparations.—*College and Clinical Record.*

---

### THE MATTEI TREATMENT.

The following from the London letter to the New York *Medical Record* is news that was to have been expected. Fortunately a more rational course was pursued in dealing with the subject than is usual in such matters, a committee of medical men having been appointed to give the remedies a fair and scientific test: The Mattei bubble has at last burst. The Mattei treatment was boomed into notoriety some months ago by Lady Paget, who wrote an article in its favor in a review, which was followed by another by Mr. Stead in his organ, the *Review of Reviews.* Mr. Stead took up the subject with his usual enthusiasm, and proposed that the value of the Mattei treatment should be properly examined by the exhibition of the Mattei remedies, to test cases of cancer under medical supervision. A medical committee was accordingly formed, and the result is what might have been expected. The patients selected as test cases have gradually got worse, no case being cured or even markedly benefited. The bubble has finally burst by the withdrawal of the Matteists from the experiment.—*Gaillard's Medical Journal.*

---

CHARCOT says: "Every drop of seminal fluid of a drunkard contains the germs of all the neuropathies."

### PHIMOSIS AND CIRCUMCISION.

My observation, experience and mature deliberation have led me to conclude in reference to this matter:

1. That each male child should be examined at birth for obstructive phimosis when we examine for imperforate anus.

2. That the prepuce should be retracted fully, and all adhesions to the glans broken up, all smegma be removed, and the nurse be instructed to retract, wash and anoint the parts daily for two weeks and once each week thereafter.

3. That a long and redundant foreskin, though freely retractable, may be a cause for local and reflex diseases, may occasion, or at least exaggerate, an undue sexual appetite, and so predispose to masturbation, sexual excesses, and ultimately to venereal diseases.

4. That such cases should be circumcised, as should the cases where the foreskin is not retractable.

5. That routine circumcision of all male infants is to be condemned. That the prepuce plays an important part in the human economy, and should not be amputated except for good and sufficient reasons.

6. The unhappy experience of the fatal case cited demonstrates that this little operation may be attended by grave dangers, and should be undertaken with this risk in view, and only after we are assured that the parents' families are not bleeders.

7. That infants may indure a copious hemorrhage far better than we have heretofore supposed.—DR. WETHERILL, *Univ. Med. Magazine.*

---

### TREATMENT OF PERSISTENT VOMITING IN PREGNANT WOMEN.

M. Neichtoube, of St. Petersburg, has frequently been able to control the persistent vomiting of pregnancy by the use of cocaine, according to the following formula: Sulphate of cocaine, 1 gm.; distilled water, 60 gm. Dose, 10 drops, to be repeated at the end of an hour if the vomiting has not ceased, and again after three hours if the desired result be not obtained. On the following day five or six drops three times a day may be given and continued until complete recovery. If the smaller dose be not sufficient to check the paroxysms, continue to use ten drops. Tampons coated with vaseline, containing two per cent. of cocaine, should be placed in the vagina and a mustard plaster or blister on the stomach. Lumps of ice may be taken internally. From our knowledge of the physiological action of vomiting and the physiological action of cocaine, the author concludes that the persisting vomiting of pregnant women is caused by the continued irritation of the bulbous center which controls the vomiting; that the irritation extends to the cerebellum, the seat of physic sensibility; that cocaine acts upon the irritated points; on the nervous center (bulb), on the terminations of the sensitive nerves (the cardiac region), and upon the cerebellum, by modifying the physic functions.—*Journal de Medicine.*—*Practice.*

---

### RULES FOR ABDOMINAL SURGERY.

Dr. J. E. Summers, Jr., Professor of Surgery in the Omaha Medical College, lays down these seven rules (*Medical Mirror*) for abdominal operations :

1. Never operate upon a practically moribund patient whose disease is chronic.

2. The same rule applies to all acute diseases or injury, except where hemorrhage is the depressing or expectedly depressing cause.

3. Never hesitate to operate upon otherwise hopeless cases, if experience has proven success to have followed interference, even if only a very small percentage.

4. Use all the principles of modern antiseptic surgery before opening the abdomen, those of aseptic surgery afterward.

5. Use drainage only after irrigation, evacuate the tube frequently and remove it early.

6. No nourishment by the mouth for twenty-four hours, giving hot water in small quantities to allay the thirst.

7. Peritonitis should be treated by large doses of calomel and copious turpentine enemas. In intestinal lesions, splinting the bowel by opium may be the proper practice.—*Medical Index.*

### LIGATION OF THE FIRST PORTION OF THE SUBCLAVIAN ARTERY AND EXCISION OF SUBCLAVIO-AXILLARY ANEURISM.

Halsted (*Johns Hopkins Hospital Bulletin*) has reported the case of a colored man, fifty-two years old, who for eight months had noticed a pulsating swelling below the left cavicle, progressively increasing in size. The tumor was smooth and almost spherical, and measured sixteen inches in circumference at its base. Slight pulsation was visible and palpable. The mass appeared to be solid but elastic. No pulse could be felt at the left wrist or at any point below the aneurism. It was determined to ligate the vessel and excise the sac. An incision was made from the sternal notch to the acromio-clavicular articulation, thence down the arm to the lower border of the major pectoral muscle over the greatest convexity of the tumor. From the inner extremity of this incision a vertical incision upward, some two inches long, was made. A third incision, about four inches long, was made vertically downward from the middle of the horizontal incision. Finally, a vertical incision, an inch and a half long, was made upward at the acromio-clavicular articulation. The flaps of skin were laid back. The wall of the aneurism was found inflamed, soft, and thin. Two strong silk ligatures were applied to the artery as it emerged from the chest, and the vessel was divided between them. The aneurism was then removed in one piece. The axillary artery was ligated at the beginning of its second part. The operation consumed three and one-half hours. The wound healed perfectly. Sixty days after the operation the patient had excellent use of the related arm.—*Medical News*.

---

THE Court of Appeals of Kentucky has recently decided that syphilis, pleaded in answer to an action to recover damages for breach of promise of marriage, is a complete defence, following the decision of the Supreme Court of the State of North Carolina, in which the same defence was interposed and sustained in a similar action.—*Weekly Medical Review*.

### EXTIRPATION OF THE INITIAL LESION IN SYPHILIS.

Dr. Ed. Ehlers (*Journal of Cutaneous and Genito-Urinary Diseases*) says:

Excision of the initial lesion is capable, in certain rare cases, of preventing general syphilitic infection of the system.

All the excisions with negative results, where they have been performed twenty-four to forty-eight hours after the appearance of the chancre, prove nothing. The object is not to extirpate as soon as possible after the appearance of the chancre, but as soon as one can after the infection. All these cases have a period of incubation of twenty to thirty days.

Excision of the initial lesion, although it has not been able to prevent general infection, has an attenuating influence upon the course of the disease.

Mercurial treatment should be instituted even if during an observation of several months the patient does not present a secondary symptom. It is possible that the secondary symptoms may be so slight as to be overlooked.

Absence of reinduration of the cicatrix does not exclude the possibility of the appearance of the secondary symptoms.

Reinduration in the cicatrix signifies a negative result, and may itself be the sole symptom which follows:

Excision should be performed in as many cases as possible, without, however, promising the patient a positive result.

The wound left after excision heals like any simple wound, if the sclerosis has been radically removed. Hence, when a chancre of large size drags along and refuses to heal when conditions are favorable, it should be extirpated.—*American Lancet.*

---

### CANNABIS INDICA IN THE PAINFUL DISEASES OF WOMEN.

Dr. J. B. Mattison, of Brooklyn, praises cannabis highly as an anodyne and hypnotic. It is not a poison, there is no fatal case on record. It must be given in full doses. Small doses are stimulating and exciting, large ones sedative. It is equally useful in dysmenorrhœa especially of the spasmodic variety, and in painful chronic metritis. In many cases of uterine cancer it allays or prevents pain. It acts well in megrim, in the neuralgias, and headaches so common in anæmic women. It is a safe and excellent hypnotic in insomnia. The tincture must be given in doses of twenty to sixty minims, and of the solid extract, one-half to two grains. Farlow recommends the following suppository in dysmenorrhœa, to be introduced every night, beginning five days before the time of the period :

℞. Ext. cannabis indica,
Ext. belladon., aa. gr. j.
Ol. theobrom, ʒ j ss.

—*Chicago Medical Times.*

---

SALICYLATE OF BISMUTH FOR SUMMER DIARRHŒA.—Infantile diarrhœa may, in many instances, be easily controlled by first securing thorough evacuation of the intestinal tract by the use of a grain or two of calomel, or some castor oil, and then giving

℞. Bismuth. salicylat., gr. xx.
Mucilag. acaciæ, fʒij.
Tinct. opii. camph., f℥ij.
Elix. simplicis q. s. ad. fʒiv.

Misce. Sig.—Keep bottle in cold water or on ice; shake well before using ; give one or two teaspoonfuls every four to six hours. This is for a child of six months to a year. For other ages the quantity of bismuth may be varied. Stimulants may be given between each dose if specially indicated, blackberry brandy being good. Fifty cases treated by this method gave only two deaths—in an unusually severe epidemic.—*Medical Index.*

---

THE highest legal tribunal in Germany has decided that legal human life does not begin in the fetus until labor has set in, and that its destruction before full term is not murder. This is progressive retrogression.—*Gillard's Medical Journal.*

### PUS IN URINE—HOW TO DISCOVER ITS SOURCE.

At a recent meeting of the New York Academy of Medicine, Dr. Keyes opened the discussion on the above topic. He said that the question could be discussed in a general way. He would assume in these general discussions that no knowledge of the cystoscope or the endoscope was necessary, and only a very slight knowledge of microscopy. It is a fact that a great many physicians are not at all acquainted with what pus is. Pus is not the healthy mucous cloud that always collects in the urine ; it is not an excess of that mucus; it is not bacteria; it is not phosphates. Pus in the urine is in the form of a granular deposit, dirty white or pinkish in color, or it may have a stringy appearance which is called by some stringy mucus. There is no such thing as stringy mucus. The stringy form of pus indicates that some portion of the genito-urinary tract is in a condition of abrasion or ulceration, and that there is decomposition of urine, and with the pus mucus is present. It means catarrh of some portion of the genito-urinary tract. These shreds that occur in the urine are practically of three kinds : the linear, the tad-pole shred, and the fleecy, cotton-like thread. The linear shred, as a rule, may be presumed to come from the anterior urethra. The origin of the tad-pole shreds is limited to no particular area ; they may come from a little follicular abscess or from a granular or excoriated spot in the membranous urethra. The fleecy, cotton-like shred is generally from the prostatic sinus; if spermatozoa are present, it is more than presumptive evidence. When the pus exudes from the meatus, it is almost conclusive evidence that the trouble lies anterior to the bulbo-membrane junction, although occasionally its source is posterior to that, especially if there has been sexual excitement. In spermatorrhea and posterior urethritis a little comes out at the meatus, but a free flow of pus usually indicates that the anterior urethra is in trouble. If the question is in doubt, you can clear it up by irrigating the anterior urethra with a solution of salicylic acid and then putting in an ordinary bulbous sound, as big as will go down to the junction, and leaving it there for thirty seconds or a minute. On withdrawing the sound, if the pus has come from the anterior urethra, there will be on the shoulder of the bulb of your instrument a little soft scab, perhaps tinged with blood. In posterior urethral pus, there is no better expedient than first washing out the anterior urethra, and then making your patient urinate in two parts. Practically, the pus will be found in the first specimen, while the rest will be reasonably clear. If you suspect that the pus originates in a prostatic abscess or the seminal vesicles, you may be able to settle this by making your patient pass his water in three parts: first let him pass about one-third; then put your finger into his rectum and press upon the seminal vesicle or the prostatic abscess, if you can appreciate it; by milking the focus of suppuration in this way you will find the discharge of pus in the second flow of urine. The third flow will be comparatively clear.

The great trouble is to distinguish between bladder pus and kidney pus. The characteristics of the two, however, are very different. If the pus has its source in the kidney, it is not flocculent; it is cheesy and heavy, and does not float around like pus from the bladder. It settles down to the bottom of the vessel. Bladder pus, on the other hand, is stringy in appearance. Urine containing bladder pus will quickly decompose; when passed it is frequently ammoniacal or neutral. In pyelitis the pus will often come in urine that is over acid, and it will remain clear and acid perhaps for a week. When blood and pus are both present in the urine, the blood will fall later than the pus, and will remain as a little thin layer on top of it.

Another distinguishing consideration is that the amount of albumen in urine is relatively greater when the pus present comes from the kidneys than it is when it comes from the bladder. If the amount of albumen is relatively high, then the probabilities are that the pus comes from the kidneys. However, if you have an ulcerated surface in the bladder, caused by cancer or stone, then you may get a considerable amount of albumen with the pus, but under ordinary circumstances the above statement holds good. If the urine contains a lot of granular pus, with perhaps one-half per cent. of albumen (by weight) you may be almost certain that you have a case of pyelitis.

Another exceedingly good way to differentiate these two conditions is by washing the bladder. You first have your patient pass his water in two parts, each of which is opaque. Then pass a soft catheter into the bladder; if you find that he is not able to empty his bladder completely, it is presumptive evidence of cystitis. Through the catheter wash the bladder with a salicylic acid solution until it is perfectly clean. Let the patient rest for an hour and then urinate; if the urine is still very cloudy, then it is probable that the pus comes from the kidney.—*The American Practitioner and News.*

---

### DOES CHLOROFORM PROMOTE POSTPARTUM HEMORRHAGE?

In this paper the author, while pointing out the great advantage of chloroform in obstetric practice, drew attention to the fact that it is not so frequently employed by accoucheurs as might have been expected. In explanation of this, two possible reasons were assigned and discussed ·

1. The deterring effect produced on the profession by the alarming and disastrous results which have too often followed the use of chloroform in surgical practice.

2. Its alleged effect in promoting postpartum hemorrhage.

In support of this view he discussed the two arguments usually put forward. These are—

*a.* The teaching of authorities.

*b.* Personal experience.

In reference to (*a*), he pointed out that there is no unanimity among teachers on this point, and hence the question can only be settled by (*b*), personal experience and observation. There is a great fallacy in arguing that because chloroform is given in a case and hemorrhage follows, that this is necessarily cause and effect,—the *post hoc ergo propter hoc* style of reasoning. Dr. Byers argued that the great majority of cases of alleged flooding after delivery, occurring when the anæsthetic was used, can be explained as being due *not* to the chloroform, but either to rapid delivery, or to a want of proper management of the third stage of labor, or to a combination of both these causes. Those who take the other view—that chloroform promotes flooding—must, when they report cases, give all the details of them, such as the age of the patient, the number of previous confinements, the extent to which the anæsthetic was given, the cause for which it was employed, the plan of delivery, and the management of the third stage of labor.

In conclusion the author gave his own experience of an extensive and increasing employment of chloroform in obstetrics, which he thought, warranted him in concluding that this anæsthetic has no effect in causing postpartum hemorrhage, provided that in all cases care is taken not to effect delivery too rapidly, and also to manage the third stage of labor according to modern teaching. In other words, if proper care is used, postpartum hemorrhage will not occur more frequently when chloroform is used than when the anæsthetic is not given.—DR. JOHN W. BYERS in *Therap. Gazette.*

---

### PRURITUS.

The following prescription comes from Allingham, and which he recommends very highly:

℞. Magnesia sulph., ʒ j.

Magnesia carb., gr. v.

Vini colchici, ♏ v.

Syr. sennæ, ʒ j.

Tr. cardam. comp., ℥ ss.

Ext. inf. chiratæ, ℥ j.

M. Sig.—Twice daily.

The mineral waters will be found useful in many cases for the same purpose.

In speaking of the medical treatment of pruritus, I will say that there are a great many prescriptions highly recommended for its local treatment, but I must confess that I know of no specific; but much relief from the itching can be obtained from the use of different remedies. A good lotion to apply after bathing the anus at night, and one which affords much relief, is composed of:

℞. Soda biboratis, ʒ ij.

Morphine hydrochlor., gr. xvij.

Acidi hydrocyanici dil., ℥ ss.

Glycerinæ, ℥ j.

Aqua ad. q. s., ℥ viij.

M. Sig.—Use as a lotion.

I have found the following glycerole to be both cooling and palliative. It is composed of:

℞. Pulv. alum, ʒ ij.

Hydrarg. chlo. mite, ʒ j.

Glycerine, ℥ iij.

M. Sig.—Paint the affected parts with it every night.

The yellowish wash and chloroform ointment I have found serviceable. One of the best formulas for the relief of the itching that I have ever used comes from Dr. Bulkley, of New York, and is composed of:

℞. Unguent picis, ℥ iij.

Unguent bellad., ʒ ij.

Tr. aconite rad., ʒ ss.

Zinci oxidi, ʒ j.

Unguent aqua ros., ʒ iij.

M. Sig.—Apply night and morning.

Pil. "Walker."—Walker's Pills.—

℞. Ext. nucis vomicæ,

Ext. belladonnæ alc., aa gr. v.

Ferri sulphat. exsicc.,

Ext. aloes, aa gr. x.

Mix. Divide into 20 pills.—*Dr. H. F. Walker.—Ex.*

---

### TYPHOID, AND "TYPHO" MALARIAL FEVER.

Comegys (C. G.) says: I am very fond of the antipyretics. I would give baths very freely if it were not for the inconvenience. A patient, however, should never be put directly into a cold bath; begin with warm water and cool down, taking care of the head by pouring cool water over it. I give the antipyretics *pro re nata*, trying to keep the temperature down to [illegible] The use of the antipyretics is sedative and diaphoretic, and increased tone will be given to the arterial circulation.

It is important that the toxic elements should be eliminated from the blood, hence the value of diaphoresis. It is not good practice to whip up the heart with stimulants, when often all that is necessary is to unload the blood of its toxic principles through the skin.

I am always looking out for hemorrhage in such cases. It is a very serious complication. I have seen the value of castor-oil purgations for the relief of intestinal hemorrhage. I tell the family that if any blood appears to begin the use of castor oil, a tablespoonful every hour, until it appears in the stools. For severe epistaxis nothing, in my experience, is equal to injections of water, as hot as can be borne, into and through the nostrils, until it ceases.

I give stimulants for their tonic effect. One to three teaspoonfuls every three hours, especially with phenacetin or acetanilid to prevent cyanosis.

If the tympanites be very great, I have a cold pack put around the abdomen, which increases the tone of the intestinal muscles, and the gas is expelled.

If symptoms of meningitis show themselves I direct that cold water be poured from a height of two feet over the face and scalp, for ten or fifteen minutes at a time, and I give ten or fifteen grains of potassium iodide in lemonade, every four hours, until the condition is ameliorated.—*Med. Standard.*

---

### SURGERY, COMPRESSION OF THE BRAIN AND THE PROVINCE OF THE TREPHINE.

Briggs (*Nashville Journal of Medicine and Surgery*, August, 1892), remarks that the term compression of the brain is not scientifically correct, but has been accepted by the profession to express a condition characterized by a group of symptoms which follow injuries, and sometimes diseases of the brain.

It is a great question with many surgeons whether compression compresses. Great pressure is often exerted on the brain structure without any symptoms indicative of compression; while on the other hand, injuries which do not induce compression, often cause the symptoms indicative of that condition.

If pressure be fixed and stationary, the brain will accommodate itself to it, and the symptoms, if any have existed, will pass off.

Injuries, which do not compress the brain, such as contusions, and lacerations may be followed by symptoms of compression.

Compression from depressed fragments of a fractured skull, is considered by Hutchinson to be an "imaginary state," and Briggs also says that he has never seen a case in which there was definite reason to think the depression produced the symptoms. The chief element of danger in depressed fractures is not compression of the brain, but irritation set up by displaced fragments of bone. The author is of the opinion that the use of the trephine should be confined to those cases where there is irritation, produced by spiculæ of bone, which press on the surface of, and often penetrate the membrances.—*Ex.*

---

### BANDAGE AFTER CATARACT OPERATION.

Dr. A. W. Calhoun still favors the old method. He says: A great deal has been said in regard to the bandage used after operating. Shall the patient sit up with the unoperated eye uncovered? The old method has proven a satisfactory one, and while it keeps the patient in bed a little longer, that weighs nothing in the balance when you consider the fact that under any other treatment you are apt to have accidents. The plan is advocated of simply putting an adhesive plaster across the closed lids; but the safest plan is to take a piece of absorbent cotton, moisten it with white vaseline, and fill out the cavity of the orbit above the closed lid, and use the flannel roller bandage, an inch and a half wide, put on in the figure of-eight position. You can put that on lightly so as to exercise no pressure upon the eye-balls, and the patient can move his head about on the pillow. I would advise those of you who have not had much experience, to see your patient twice a day. Take off the bandage, and with bichloride of mercury bathe the lid on the outside, but do not open the lids unless there is decided inflammation. I keep up that process of twice a day dressing the eye for four or five days and then lengthen the interval. After the patient has been in bed four to six days I turn one eye loose and keep the other bound until it is ready to be turned loose permanently. That is my mode of after treatment so far as the bandage is concerned.—*Virginia Medical Monthly.*

---

### INTESTINAL FLUXES, ETC.

IN a practice of over twenty years I have never met with anything the equal of this pill to check fluxes of the bowels, such as dysentery, diarrhœa, bloody flux, cholerine, intestinal tuberculosis, etc., and believing it to be superior to all other similar remedies vaunted for cholera, herein make it known:

℞. Pulv. opii.,
Pulv. plumb. acetat.,
Camphor gum, āā gr. xxx.
Fl. ext. capsicum, gtt. x.
Beechwood creosote, gtt. v.
Alcohol q. s. to dissolve camphor.

M. F.—Pills No. 30.

Sig.—One to 6 pills a day, according to the urgency of the case.—J. ZACK TAYLOR, M. D., in *Times and Register.*

---

### CYSTITIS.

Cystitis is treated by Professor Bangs by washing out the bladder with borosalicylic solution, and injecting twice daily ʒj to ʒiij of the following iodoform emulsion, which is much used at St. Luke's:

℞. Iodoformi, ʒij.

Glycerini,

Mucil. acaciæ, āā ℥ss.

Aquæ, ad ℥viij.

Rub up the iodoform with the mucilage, then add the glycerin, and finally water.—*Med. Summary.*

### HOW TO POULTICE THE EAR.

Poulticing an ear may seem to be a simple operation, but there is, nevertheless, a right and a wrong way of doing it, and it appears that the wrong way is the one usually adopted. At least so says Dr. Albert H. Buck, of New York, in an article on aural therapeutics in the March number of the new *International Medical Magazine.* Dr. Buck says that while heat is one of the best remedies in painful inflammations of the middle ear and the poultice is one of the best methods of applying heat, as usually put on the poultice has little effect. What should be done, he says, is first to fill the external auditory canal with lukewarm water, the head resting on the unaffected side upon the pillow. Then a large flaxseed poultice is applied over the ear as hot as can be borne. The column of water is thus kept warm and acts as a conductor of heat between the poultice and the inflamed surface.—*N. W. Lancet.*

---

### TO DISGUISE THE TASTE OF TURPENTINE.

For this purpose Dr. H. C. Wood in the *Therapeutic Gazette*, recommends glycerine as in this formula:

℞. Olei terebinthinæ, fl. ʒij.

Glycerine, fl. ℥j.

Mucil. acaciæ, fl. ℥iss.

Aquæ menthæ piperitæ, q. s. ad. ℥viij.

M. Sig.—Tablespoonful every four hours during the day. Shake well.

---

### FOR HEMORRHOIDS.

The *Rev. de Therap. General* recommends:

℞. Atropinæ sulphat., gr. iv.
Acid. tannic, gr. vj.
Morphinæ sulphat., gr. vj.
Cocainæ hydrochlorat., gr. xxx.
Vaselin, ℥j.

Fiat unguentum.

M. Sig.—Apply a small quantity to the hemorrhoid after each stool.—*Medical and Surgical Reporter.*

### FISTULA IN ANO.

As far as diagnosticating fistula in ano is concerned, that is quite an easy matter, but to tell exactly the character of the fistula we have to deal with is quite another thing. An operation that will cure one fistula will not cure another. Therefore, no general rule will apply to these cases. There are several things to be taken into consideration in properly diagnosticating or prognosticating a case of fistula.

1. Is it a simple fistula, and has it but one channel ?
2. Is it a progressive or non-progressive fistula ?
3. Is it due to any special diathesis, as tubular, syphilitic, etc ?
4. Does it exist as a disease *per se*, or is it the result (secondary) of stricture.

These are essential considerations, and will decide the method of operating and after-treatment, and the prognosis

To illustrate : If the case is one of simple fistula with but one channel, a single division of tissue, either by knife or ligature, will effect a cure.

If it is a progressive fistula with a great discharge of pus and a rapid breaking down of tissue, an operation *by the knife* should be advised at once. If it is non-progressive no hurry need be had, and the patient can adapt himself to circumstances.

If the disease is due to any special diathesis, such diathesis must be ascertained in order that the proper medical as well as surgical treatment can be afforded. Indeed, upon this is decided whether an operation is warranted at all.

If the fistula be secondary to a stricture of the bowel, no operation is permissible until the strictured condition is righted.

I consider, therefore, that these are points of much more significance than to determine whether the fistula be an external, internal, or complete one. It has often occurred to me that the authorities put too great stress upon this division of fistulæ. It matters very little to the surgeon who is going to operate, which variety he is going to deal with, for he is going to do pretty much the same operation in all. It is the complications that concern him, not this kind of division.—DR. JOSEPH M. MATHEWS in *International Journal of Surgery.*

### PHTHISIS.

Repeated testimony has been borne by almost every observer as to the undoubted efficacy of cod liver oil and the hypophosphite of lime in the early stage of phthisis. The former gives nourishment to the body, and the latter exercises an alterative influence upon the process of tubercular change. Yet cod liver oil should not be given by routine, but with definite rules as to its administration. To secure proper assimilation, it should be prescribed in a teaspoonful dose at bedtime for three successive nights. Then a dessertspoonful dose after dinner and at bedtime, andaf terwards in a tablespoonful dose after each meal. If so prescribed it does not cause eructation or nausea, and the doses may be increased in accordance with the wishes of the patient until the end of the fifth week, when it should be stopped for a week, and resumed. With each dose of the oil five grains of hypophosphite of lime, dissolved in a little hot water, should be taken. Thus administered, these medicines notably increase the strength of the patient, and returning health is evidenced by increased weight. If circumstances permit, and if the patient be young, a sea voyage is of inestimable advantage in maintaining the progress made to recovery, and if the voyage be to New Zealand and back, in many cases the patient's health is established. Charteris firmly believes that the hypophosphite of lime is the only hypophosphite of any value in tubercular disease. This opinion was expressed by him in communications to the *Lancet* in 1876, when this salt was first introduced to the profession as an efficient therapeutic agent. All his subsequent experience confirms this view, and he does not consider its influence is increased by its combination with the sodium and iron salts.—*Charteris, London Lancet.—Therap. Gazette.*

---

Prof. Hare says that for fainting, as a rapidly acting stimulant, give alcohol, hot and concentrated. The hot alcohol acts much more quickly than cold, because the cold alcohol, before it could be absorbed, must be heated up to the temperature of the body. —*College and Clinical Record.*

### A SIMPLE METHOD OF DRESSING OPERATION WOUNDS.

The patient is bathed and the part to be operated upon is shaved and thoroughly scrubbed with soft soap (the green semi-transparent variety being the best), which is more efficacious in cleansing the skin then ether or turpentine ; then it is sponged with a strong corrosive sublimate solution, and protected, until the time of the operation, by a towel wrung out in sublimate lotion. An unirritating gauze, unimpregnated by antiseptics, is thus prepared—a soft unmedicated gauze, costing one penny per yard for one thousand yards, is rendered aseptic by being kept in one in five hundred corrosive sublimate solution in earthenware jars. The solution is changed once a fortnight, and the gauze becomes whiter and softer as time goes on; when required a sufficient quantity is cut off, wrung as dry as possible, and washed free of the mercuric chloride in a sterilized basin of boiling or recently boiled water. The operator takes it out of the basin, wrings it dry, shakes it loosely and dresses his wound. The moisture should be as completely expelled as possible. The filtration of the air and pressure on the wound is accomplished by some unirritating aseptic absorbent cotton wool, such as salicylic wool, or cheaper ordinary absorbent wool, which has been sterilized by super-heated steam, and kept in stoppered glass bottles. It is claimed for this method that it is efficient (suppuration being rarely met with, even in out-patient cases), economical (the sublimate having an almost nominal cost), and extremely simple.—HUBERT HIGGINS, in *British Medical Journal.*

---

At the recent meeting of the American Electro Therapeutic Association, held in New York, the following officers were elected for the ensuing year :

President—Dr. Augustus H. Goelet, of New York,

Vice-Presidents—Dr. W. F. Hutchinson, of Providence, R. I.; Dr. W. J. Herdman, of Ann Arbor, Michigan.

Secretary—Dr. M. A. Cleaves, of New York.

Treasurer—Dr. R. J. Nunn, of Savannah, Ga.

Executive Committee—Dr. W. J. Morton, of New York ; Dr. G. Betton Massey, of Philadelphia; Dr. Robert Newman, of New York ; Dr. Charles R. Dickson, of Toronto, Canada; Dr. J. H. Kellogg, of Battle Creek, Michigan.

The next meeting will be held in Philadelphia September 12, 13 and 14, 1893.

